# Medication utilization pattern for management of pregnancy complications: a study in Western Nepal

**DOI:** 10.1186/s12884-016-1068-8

**Published:** 2016-09-20

**Authors:** Ramesh Devkota, G. M. Khan, Kadir Alam, Amisha Regmi, Binaya Sapkota

**Affiliations:** 1 Department of Drug Administration, Kathmandu, Nepal; 2 Pokhara University, Kaski, Nepal; 3 Manipal Teaching Hospital, Kaski, Nepal; 4 Tribhuvan University Teaching Hospital, Kathmandu, Nepal; 5 Nobel College, Kathmandu, Nepal

**Keywords:** Pregnancy, Complication, Management, Drug utilization, Nepal

## Abstract

**Background:**

Drugs used during pregnancy can adversely affect the health and life of the mother and unborn child. However, the fact that drugs are needed to mitigate complications during pregnancy cannot be avoided. The present study was designed to identify the common complications during pregnancy and assess the medications that have been used to mitigate those complications in an attempt to improve drug prescribing during pregnancy.

**Methods:**

A hospital based cross sectional study was conducted at Manipal Teaching Hospital, Nepal in 275 pregnant women presenting with at least one complication and the drugs prescribed for the management of those complications were analyzed.

**Results:**

Majority of the patients in this study were in the age group 20–24 (44 %) and in the third trimester (53.8 %). Maximum patients complained pain (back, abdominal, lower abdominal, neck, pelvic) as primary complication (24.3 %) which was followed by nausea/vomiting, upper respiratory tract complications, acid reflux disease and others. Of the total prescriptions eighty six (86) did not have any medicines prescribed to the patients except multivitamins and nutritional supplements. The average drugs prescribed per patient was 2.78 in outpatient setting and 5.41 in in-patients. Ranitidine, hyoscine butylbromide, paracetamol were the most frequently prescribed medications. Antimicrobials comprised 12.8 % of total drugs prescribed and 18 % of total drugs were fixed dose combinations. Two hundred and thirty four (234) prescriptions out of 275 were prescribed by brand names. Most of the prescribed drugs were from FDA pregnancy category B and C.

**Conclusion:**

The present finding showed that pregnant patients were prescribed medications almost only when necessary and those considered safe during pregnancy were chosen to a large extent. However, few teratogenic drugs (2.49 % of total drugs prescribed) were also found to be prescribed which might need further assessments.

**Electronic supplementary material:**

The online version of this article (doi:10.1186/s12884-016-1068-8) contains supplementary material, which is available to authorized users.

## Background

Pregnancy is associated with a wide range of pharmacokinetic [1, 2] and physiological [3, 4] changes. Those changes quite often present physiological complications during pregnancy. Apart from this, pregnant women also present with other concurrent medical illness [5, 6] and chronic diseases. Irrespective of the risk of drug therapy during pregnancy, drug use cannot be avoided in conditions where benefits potentially outweigh the risks involved [7]. The risks associated with drug use in pregnancy can be two fold: incidence of adverse effects to the mother followed by teratogenic effects to the growing fetus [8, 9]. This demands that a careful consideration is required before drugs are prescribed to pregnant patients.

It is also a common practice to use iron, calcium, multivitamins, folic acid and other nutritional supplements during pregnancy to replenish for any nutritional deficiencies. Use of herbal and homeopathic supplements/medicines is also common [10]. Many researches have been conducted worldwide for assessing the occurrence of complications and drug utilization patterns [5–7, 11–13] during pregnancy despite of which safety of each drug has not been established. Although rare, studies demonstrate the prescription and use of drugs from FDA category X during pregnancy [6, 11–13].

Nearly 18.5 % of the diseases among women of reproductive age are related to pregnancy related complications [14] and deaths due to those complications is higher in developing nations. Nepal has a maternal mortality ratio of 170 per 100,000 live births [15] which is one of the worst figures in south Asia. Postpartum hemorrhage, eclampsia, infections, obstructed labor, complications of abortions are the major causes of maternal mortality in Nepal [14]. More than 42 % of the adult population in Nepal is illiterate and when compared to sex ratio, it is higher among females [16]. This literally means that pregnant women would know less about medications prescribed to them which demands for effective rational prescribing. Moreover, there are limited researches in medication use during pregnancy in Nepal for which we decided to carry out this study. If the common complications during pregnancy were to be known, healthcare facilities would try to accommodate necessary resources in advance so that the problems could be dealt with ease. This knowledge used alongside rational prescribing which taken into account for the low literacy rate of Nepal could prevent any un-intended drug induced risk to the mother and child. Thus this study was carried out with the aim to find out the type and nature of common pregnancy complications (arising either due to physiological changes or any other associated medical illness) in Western Nepal and access the medications used to mitigate those complications in an attempt to improve the drug prescribing patterns during pregnancy.

## Methods

### Study design, site, sample size and duration of study

This was a hospital based cross-sectional study, carried out at Obstetrics and Gynecology (OBG) Ward of Manipal Teaching Hospital, Fulbari, Kaski, Nepal from July to December 2013. Manipal Teaching Hospital is a 750 bed community based tertiary care hospital located at the Western Region of Nepal. A total of 275 prescriptions of pregnant women with various complications were included in this study.

### Ethical consideration

The study was ethically approved by Manipal Teaching Hospital, Kaski, Nepal. Informed consent was taken from the patients at the time of enrollment in the study.

### Sampling technique and Inclusion criteria

Simple purposive sampling technique was used to select the subjects for the study. The pregnant women who visited the hospital during the study duration and presented with at least one newly diagnosed complication were included for the study. Any associated physiological, medical, obstetrical, gynaecological condition identified by the consulting physician as an illness and mentioned in the prescription of the patient has been considered as a complication in this study. Any patient coming for follow-up was not included in the study to avoid the repetition of prescriptions. Hence, 275 prescriptions represent 275 individual patients with complications. Both out-patient and in-patient prescriptions were studied. In case of admitted patients, the prescriptions were studied only once. All patients approached responded to us and thus the response rate was 100 %.

### Exclusion criteria

Pregnant women who did not present with any complications and those who had complications before the time of study duration were not included in the study.

### Data collection

Characteristics related to patient demographics, complications and medicines used for the treatment of those complications were collected in the proforma designed for the same. Medications prescribed by the physicians at the time of consultation were included in the study. Data on self medications and/or any other medication being taken besides those prescribed at that point of time were not collected.

### Statistical analysis

Medicines were classified according to the Anatomic Therapeutic Chemical (ATC) and US Food and Drug Administration (FDA) risk classification. Data were analyzed by statistical package for social sciences (SPSS) software version 22. Descriptive statistics, in the form of frequency distribution and associated percentage, were employed for the presentation of complications and medicines used for their management. Associations among complications, age and trimester of the respondents were analyzed by Chi square test and/or Fisher’s exact test (where the individual cell frequencies were less than 5). The *p* value <0.05 was considered statistically significant at 95 % confidence interval.

## Results

### Demographic details

Majority of the patients in the study were from the age group 20–24 (44 %), which was followed by age group 25–29 (32.7 %), 15–19 (12.4 %), 30–34 (9.8 %), 35–39 (0.7 %) and 40–44 (0.4 %) respectively. According to trimester distribution, 53.8 % of the populations were from third trimester. This was followed by women from second trimester (26.9 %) and only 19.3 % from first trimester.

### Complications observed

A total of 275 complications were observed which are presented in Table 1.

**Table 1:** Frequency distribution of complications

Complication		1st trimester (%)	2nd trimester (%)	3rd trimester (%)	Total (%)
Alimentary tract and metabolism (*n* = 73)	Acid reflux disease	2 (0.7 %)	8 (2.9 %)	10 (3.6 %)	20 (7.3 %)
	Constipation	0	2 (0.7 %)	2 (0.7 %)	4 (1.5 %)
	Diabetes mellitus	0	1 (0.4 %)	0	1 (0.4 %)
	Diarrhoea	1 (0.4 %)	1 (0.4 %)	1 (0.4 %)	3 (1.1 %)
	Hyperemesis gravidarum	5 (1.8 %)	0	0	5 (1.8 %)
	Irritable bowel disease	0	0	1 (0.4 %)	1 (0.4 %)
	Loss of appetite	0	2 (0.7 %)	0	2 (0.7 %)
	Nausea/Vomiting	15 (5.5 %)	17 (6.2 %)	8 (2.9 %)	40 (14.5 %)
Blood and blood	Anemia	1 (0.4 %)	0	0	1 (0.4 %)
Forming organs (*n* = 16)	PV bleeding	5 (1.8 %)	0	9 (3.3 %)	14 (5.1 %)
	Thrombocytopenia	0	0	1 (0.4 %)	1 (0.4 %)
Cardiovascular (*n* = 6)	Pre-eclampsia	0	0	6 (2.2 %)	6 (2.2 %)
Dermatologicals (*n* = 7)	Itching	0	2 (0.7 %)	5 (1.8 %)	7 (2.5 %)
Endocrinological (*n* = 6)	Hypothyroidism	1 (0.4 %)	0	4 (1.5 %)	5 (1.8 %)
	Pituitary adenoma	0	0	1 (0.4 %)	1 (0.4 %)
Genito-urinary (*n* = 20)	PV discharge	0	0	8 (2.9 %)	8 (2.9 %)
	Urinary tract infection	5 (1.8 %)	6 (2.2 %)	8 (2.9 %)	19 (6.9 %)
	Anxiety	0	1 (0.4 %)	1 (0.4 %)	2 (0.7 %)
	Depression	0	1 (0.4 %)	0	1 (0.4 %)
Nervous (*n* = 85)	Epilepsy	1 (0.4 %)	0	1 (0.4 %)	2 (0.7 %)
	Fever/Headache	2 (0.7 %)	2 (0.7 %)	6 (2.2 %)	10 (3.6 %)
	Pain*	0	15 (5.5 %)	52 (18.9 %)	67 (24.4 %)
	Psychosis	0	1 (0.4 %)	0	1 (0.4 %)
	Tingling sensation	1 (0.4 %)	1 (0.4 %)	0	2 (0.7 %)
	Asthma	0	2 (0.7 %)	1 (0.4 %)	3 (1.1 %)
Respiratory (*n* = 29)	Pneumonia	0	0	1 (0.4 %)	1 (0.4 %)
	Tuberculosis	1 (0.4 %)	0	0	1 (0.4 %)
	Upper respiratory infections**	5 (1.8 %)	6 (2.2 %)	13 (4.7 %)	24 (8.7 %)
Sensory (*n* = 1)	Dry eye	0	1 (0.4 %)	0	1 (0.4 %)
	Edema	1 (0.4 %)	3 (1.1 %)	5 (1.8 %)	9 (3.3 %)
	Loss of pregnancy	7	0	2 (0.7 %)	9 (3.3 %)
Various (*n* = 32)	Oligohydramnios	0	0	1 (0.4 %)	1 (0.4 %)
	Polyhydramnios	0	0	1 (0.4 %)	1 (0.4 %)
	Weakness	0	2 (0.7 %)	0	2 (0.7 %)
Total		53 (19.3 %)	74 (26.9 %)	148 (53.8 %)	275 (100 %)

*total 67 pain complications seen were: abdominal pain (14), back pain (18), lower abdominal pain (30), neck pain (2), pelvic pain (3)

**upper respiratory tract infections consisted of cough cold and sore throat

The frequency distribution of the complications revealed that 67 pregnant women (24.4 %) suffered from various types of pain (back, abdominal, lower abdominal, neck, pelvic pain), 40 (14.5 %) from nausea/vomiting, 20 (7.3 %) from acid reflux disease, 24 (8.7 %) from upper respiratory tract infection, 14 (5.1 %) from per vaginal bleeding and 19 (6.9 %) from urinary tract infection.

Of the total patients 75 (27.3 %) needed hospitalization. Common reasons for hospitalization include abdominal pain (*n* = 8), anemia (*n* = 1), diabetes mellitus (*n* = 1), edema (*n* = 1) epilepsy (*n* = 1), fever/ headache (*n* = 6), hyperemesis gravidarum (*n* = 5), hypothyroidism (*n* = 1), loss of pregnancy (*n* = 9), lower abdominal pain (*n* = 3), oligohydraminos (*n* = 1), pneumonia (*n* = 1), polyhydraminos (*n* = 1), preeclampsia (*n* = 6), PV bleeding (*n* = 8), PV discharge (*n* = 5), thrombocytopenia (*n* = 1), urinary tract infections (*n* = 12) and upper respiratory tract infections (*n* = 4) .

### Association of complications with age of respondents

In 20–24 years age group, the patients were found to be suffering mainly from pain (30, 46.2 %), nausea/vomiting (17, 40.5 %), acid reflux disease (12, 60 %), urinary tract infection (10, 52.6 %) and per vaginal bleeding (9, 64.3 %). In age group 25–29 years, major complications were pain (19, 29.2 %), nausea/vomiting (14, 33.3 %), and upper respiratory tract infection (11, 45.8 %). PV bleeding, urinary tract infections, preeclampsia, loss of pregnancy were seen in other age groups too. The complications were not significantly associated with the age of the patient (*p* 0.988) (Table 2).

**Table 2:** Association of complications with the age of the respondents

Complications	Age of the respondents (in years) (n,%)						Total	*p* value
	15–19 years	20–24 years	25–29 years	30–34 years	35–39 years	40–44 years		
Acid reflux disease	1 (5)	12 (60)	5 (25)	2 (10)	0	0	20 (100)	0.988
Anemia	0	1 (100)	0	0	0	0	1 (100)	
Anxiety	0	1 (50)	0	1 (50)	0	0	2 (100)	
Asthma	0	2 (66.7)	1 (33.3)	0	0	0	3 (100)	
Constipation	0	2 (50)	2 (50)	0	0	0	4 (100)	
Depression	0	0	0	1 (100)	0	0	1 (100)	
Diabetes mellitus	0	0	0	1 (100)	0	0	1 (100)	
Diarrhea	0	0	2 (66.7)	1 (33.3)	0	0	3 (100)	
Dry eye	0	1 (100)	0	0	0	0	1 (100)	
Edema	0	5 (55.6)	2 (22.2)	1 (11.1)	1 (11.1)	0	9 (100)	
Epilepsy	0	2 (100)	0	0	0	0	2 (100)	
Fever/Headache	3 (30)	4 (40)	3 (30)	0	0	0	10 (100)	
Hyperemesis gravidarum	0	2 (40)	3 (60)	0	0	0	5 (100)	
Hypothyroidism	0	3 (60)	1 (20)	1 (20)	0	0	5 (100)	
Irritable bowel disease	0	0	1 (100)	0	0	0	1 (100)	
Itching	1 (14.3)	1 (14.3)	4 (57.1)	1 (14.3)	0	0	7 (100)	
Loss of appetite	0	0	1 (50)	1 (50)	0	0	2 (100)	
Loss of pregnancy	2 (22.2)	3 (33.3)	2 (22.2)	2 (22.2)	0	0	9 (100)	
Nausea/Vomiting	9 (22.5)	16 (40)	14 (35)	1 (2.5)	0	0	40 (100)	
Oligohydramnios	0	0	1 (100)	0	0	0	1 (100)	
Pain	8 (11.9)	31 (46.3)	19 (28.4)	8 (11.9)	0	1 (1.5)	67 (100)	
Pituitary adenoma	0	0	1 (100)	0	0	0	1 (100)	
Pneumonia	0	0	1 (100)	0	0	0	1 (100)	
Polyhrdramnios	0	0	1 (100)	0	0	0	1 (100)	
Preeclampsia	1 (16.7)	2 (33.3)	2 (33.3)	1 (16.7)	0	0	6 (100)	
Psychosis	1 (100)	0	0	0	0	0	1 (100)	
PV bleeding	1 (7.1)	9 (64.3)	2 (14.3)	2 (14.3)	0	0	14 (100)	
PV discharge	1 (12.5)	4 (50)	3 (37.5)	0	0	0	8 (100)	
Thrombocytopenia	0	0	1 (100)	0	0	0	1 (100)	
Tingling sensation	0	1 (50)	0	1 (50)	0	0	2 (100)	
Total	34 (12.4)	121 (44)	90 (32.7)	27 (9.8)	2 (0.7)	1 (0.4)	275 (100)	
Tuberculosis	0	0	1 (100)	0	0	0	1 (100)	
Upper respiratory tract infection	5 (20.8)	7 (29.2)	11 (45.8)	1 (4.2)	0	0	24 (100)	
Urinary tract infection	1 (5.3)	10 (52.6)	6 (31.6)	1 (5.3)	1 (5.3)	0	19 (100)	
Weakness	0	2 (100)	0	0	0	0	2 (100)	

### Association of complications with trimester of respondents

Majority of the patients suffered from various complications in the third trimester. Besides pain, (52, 77.6 %), upper respiratory tract infection (14, 58.3 %) and acid reflux disease (10, 50 %) were the most frequently encountered complications in the third trimester. In the second trimester acid reflux disease (8, 40 %), urinary tract infections (6,31.6 %) and upper respiratory tract infections (6, 25 %) were common. In the first trimester, the pregnant patients suffered mainly from nausea/vomiting (16, 38.1 %) and loss of pregnancy (7, 77.8 %). The complications were significantly associated with the trimester of the pregnancy (p 0.000) (See Additional file 1).

### Management of complications

Of the total 275 patients, 254 received a prescription for iron, calcium and folic acid. Twenty two patients also received prescriptions for tetanus toxoid injection and 34 received vitamins, nutritional supplements and other alternative medicines. Eighty-six patients were not prescribed with any medicines except multivitamins and nutritional supplements.

For the management of pain hyoscine butylbromide and diclofenac were most preferred. Likewise, paracetamol and ibuprofen for fever and headache; ranitidine, antacids and pantroprazole for acid reflux disorders; iron for anemia; alprazolam in anxiety; salbutamol and ipratropium bromide for asthma; bisacodyl and lactulose in constipation; fluoxetine in depression; insulin for diabetes mellitus; lamotrignine, carbamazepine, sodium valporate, valporic acid for epilepsy; ondasetron, metoclopramide, domperidome, granisetron as antiemetics; thyroxine for hypothyroidism; loratidine, levocetirizine, cetirizine as antiallergics; tranaxemic acid for bleeding; methyldopa, nifedipine, amlodipine, hydrochlorothiazide, magnesium sulphate in pre-eclampsia; cefixime, nitrofurantoin, amoxicillin, ampicillin, ceftriaxone, gentamicin, povidone iodine, metronidazole, piperacillin + tazobactam combination, amoxicillin + potassium clavalunate combination as anti-infectives; terbutaline, bromohexine, guanefensin, oxymetazoline for cough and cold were the commonly prescribed drugs for the management of associated complications.

The common drugs that were prescribed in different complications are presented in detail in Additional file 2.

### Distribution of drugs

A total of 961 drugs (including nutritional supplements and alternative medicines) have been prescribed to 275 patients. This consists of a minimum number zero (no drug) to a maximum of 17 drugs in one patient presenting with eclampsia. The prescribed drugs when categorized into generic class, a total of 93 different drugs have been prescribed (excluding nutritional supplements and alternative medicines). They were classified as per the WHO ATC classification system and are presented in Table 3.

**Table 3:** Classification of drugs

ATC category	1st trimester (%)	2nd trimester (%)	3rd trimester (%)	Total (%)
Alimentary tract and metabolism	44 (4.6 %)	94 (9.8 %)	211 (22 %)	349 (36.3 %)
Anti-infectives for systemic use	34 (3.5 %)	26 (2.7 %)	83 (8.6 %)	143 (14.9 %)
Antiparasitic products, insecticides and Repellants	1 (0.1 %)	0	1 (0.1 %)	2 (0.2 %)
Blood and blood forming organs	42 (4.4 %)	69 (4.4 %)	145 (15.1 %)	256 (26.7 %)
Cardiovascular system	0	0	19 (2 %)	19 (2 %)
Dermatologicals	0	3 (0.3 %)	3 (0.3 %)	6 (0.6 %)
Genitourinary system and sex hormones	13 (1.4 %)	2 (0.2 %)	7 (0.7 %)	22 (2.3 %)
Musculoskeletal system	1 (0.1 %)	2 (0.2 %)	9 (0.9 %)	12 (1,2 %)
Nervous system	13 (1.4 %)	11 1.1 %)	53 (5.5 %)	77 (8 %)
Respiratory system	6 (0.6 %)	14 (1.5 %)	16 (1.7 %)	36 (3.7 %)
Sensory organs	0	1 (0.1 %)	0	1 (0.1 %)
Systemic hormonal preparations exc.. Sex hormones and insulin	1 (0.1 %)	0	12 (1.2 %)	13 (1.4 %)
Various	5 (0.5 %)	4 (0.4 %)	16 (1.7 %)	25 (2.6 %)
Total	160 (16.6 %)	226 (23.5 %)	575 (59.8 %)	961 (100 %)

Alimentary tract and metabolism drugs were the most frequently prescribed drugs (36.3 %) which was followed by drugs from blood and blood forming organs (26.6 %). Anti-infectives for systemic use account for 14.8 % of the total drugs prescribed which the third is highest in the category.

Out of 961 drugs prescribed, iron, calcium and folic acid given for routine supplementation account for 450 drugs which is nearly 47 % of the total drugs prescribed. Besides these, ranitidine is the most frequently prescribed drug accounting for 5.5 % of the total drugs prescribed. This is then followed by hyoscine butylbromide (3.5 %), paracetamol (3.5 %), ibuprofen + paracetamol combination (3.1 %), ondansetron (2.9 %) and amoxicillin + potassium clavulanate combination (2.9 %).

The list of categorywise individual drugs have been classified in Additional file 3.

### Use of antimicrobials

A total of 18 different types of antimicrobial agents (excluding tetanus toxoid) have been used with a total number counting to 123 which comprises of 12.8 % of total drugs used. Majority of the antibiotics have been used in severe hospitalized complications. The top four antibiotics based on prescription frequency include cefixime, amoxicillin, metronidazole and ceftriaxone.

### Use of fixed dose combinations

Of the different fixed dose combinations, combination of iron + folic acid is the maximum which accounts for a total of 173 drugs out of 961. Except iron + folic acid a total of 57 fixed dose combinations have been prescribed. Of all these combinations ibuprofen + paracetamol is maximum (30 out of 57).

### Drug use per trimester

In an average 3.49 drugs have been used per patient. Trimester wise drug use per patient is maximum in the third trimester (3.88) followed by second (3.05) and first (3.01).

Regarding the fact that both in patients and out-patients cases have been considered in this study, it would be necessary to see drug use per patient in these two situations. The distribution of drugs and other parameter differences between in patients and out patients can be viewed from the Table 4. It is seen from the table that the in-patients in an average receive 2.63 more drugs than the outpatients.

**Table 4:** In patient and out patient differences

Parameter	Out patient	In-patient
Total population	200 (72.7 %)	75 (27.3 %)
Total number of drugs used	555	406
Number of drugs per patient	2.78	5.41
Complications per trimester		
First	33	20
Second	69	5
Third	98	50
Drug used per trimester		
First	79	80
Second	195	29
Third	281	297
Number of drugs per patient per trimester		
First	2.39	4
Second	2.82	5.8
Third	2.86	5.94
Mean duration of hospital stay	Not applicable	4.65 days
Use of injections	No (except tetanus toxoid)	Yes

### Generic and brand prescribing

Of the total 275 prescriptions only 41 were prescribed with generic drug names and the rest by brand names.

### FDA pregnancy category and prescription of teratogenic drugs

**Fig. 1:**
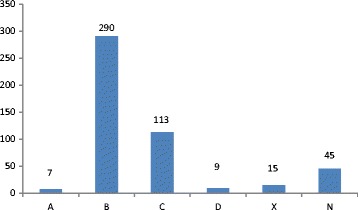
FDA pregnancy categories of drugs

For the analysis of FDA pregnancy category of drugs and identification of teratogenic drugs, we excluded the nutritional supplements, vitamins and mineral supplements. Out of 961 drugs, these included 479 drugs and the analysis was done in remaining 482 drugs. The FDA pregnancy category of drugs has been presented in Fig. 1.

Majority of the medicines prescribed were from FDA pregnancy category B (60.2 %) and C (23.4 %). 24 drugs (4.9 %) prescribed from category D (*n* = 9) and X (*n* = 15) were found to be teratogenic. Of the teratogenic drugs, progesterone was most frequently prescribed (*n* = 9) followed by misoprostol (*n* = 5), alprazolam (*n* = 2), povidone iodine (*n* = 2), carbamazepine (*n* = 1), human chorionic gonadotropin (*n* = 1), hydrochlorothiazine (*n* = 1), hydroxyprogesterone (*n* = 1), phenobarbitone (*n* = 1) and valporic acid (*n* = 1).

### Dosage form of the drugs

Of the different dosage forms of drugs tablets were the most frequently prescribed comprising of 61.4 % of the total drugs prescribed. It was then followed by capsules, injections and syrup which accounted for 21.8, 10.3 and 1.6 % of total prescribed drugs respectively. Remaining were suspension, solution, drop, lotion, gel, ointment, inhaler, granules and pressary. In the tablet dosage forms calcium is the most prescribed one comprising of 33.6 % of the total tablet dosage forms. Next to calcium are ranitidine, folic acid, iron, paracetamol, cefixime and ibuprofen + paracetamol combination each of which constitute of 7.9, 7.1, 6, 5.5, 5.2 and 5 % of total tablet dosage forms respectively. Others constitute the remaining. In the capsules section, iron + folic acid combination has the highest percentage of 82.3 % of all capsule dosage form. This is followed by amoxicillin and nifedipine in second and third places. The ascending order of injection dosage form can be considered from tetanus toxoid injection, ceftriaxone, hyoscine butylbromide, metronidazole, dexamethasone, ondansetron and ranitidine respectively each comprising of 22.2, 18.1, 11.1, 8, 7, 7 and 6 % of total drugs in injection form.

## Discussion

As during other situations, pregnant women often suffer from different types of complications. In this study we found that nausea and vomiting was the most common complication in the first and second trimester whereas back pain and abdominal pain in the third trimester which is most importantly associated with the physiology of pregnancy. Cases of hospitalizations observed were similar to a study which reports preterm labor, genito urinary complications and preeclampsia as major causes of hospitalizations [17]. Although the selection of patients was not biased in terms of trimester majority of the sampled women belonged to the third trimester. This might need further clarification; however, it can give us the idea that pregnant women frequently visited the hospital during their late trimesters.

When we observed the management of complications we found that 31.2 % of total prescriptions did not contain any drugs besides multivitamins and nutritional supplements. Hyoscine butylbromide, the most frequently prescribed drug for abdominal pain is considered relatively safer in pregnancy and normally not associated with adverse outcomes in mother or neonates [18]. Even though rest and manual therapy are suggested, acetaminophen and other NSAIDS are reported as first line pain management drugs during pregnancy [19, 20]. All antiemetics are considered relatively safe during pregnancy and among them metoclopramide and ondansetron are safer [4, 21, 22]. The use of antibiotics was seen quite frequent for both prophylaxis and treatment. Common antibiotics used here include cefixime, nitrofuratoin, amoxicillin, ampicillin, ceftriaxone, gentamycin. All these antibiotics are considered relatively safe during pregnancy of which cephalosporins and penicillins are considered safer. However, gentamycin is associated with nephrotoxicity and might need precaution before use [23].

Majority of the drugs prescribed in this study belonged to FDA pregnancy category B and C and 3 different types of drugs were from category X namely HCG, progesterone and misoprostol. Misoprostol was basically used for induction of abortion and in intra-uterine fetal death and thus it’s use could be justified. A study [11] also shows that majority of drugs taken by pregnant women were from FDA pregnancy category C (28.8 %) and B (26.5 %) and drugs from category D and X basically included progesterone and hormonal contraceptives respectively. Of the FDA pregnancy category D drugs, antiepileptics have been most frequently prescribed. Although being teratogenic majority of antiepileptic drugs still lack much safer alternative drugs [24]. Category B drugs are considered relatively safe to use in pregnancy as animal studies have failed to demonstrate any risk. However, category C drugs should be used only when potential beneficial conditions as these medicines have shown adverse effects on animal studies. This shows that category C medications prescribed here might need further assessment. However, lack of ideally safe drugs and the need to treat the condition outweigh the risks involved and a physician is compelled to prescribe those drugs. With respect to prescription of teratogenic drugs, the finding of this study was high as compared to a study conducted in Pakistan which reports 2.3 % of the prescribed drugs as teratogenic [12].

In other complications the safest drugs possible have been seen to be prescribed which reflect a positive aspect of prescribing. However, some anomalies were also seen like use of amoxicillin in itching only, use of fixed dose combinations when it might not be necessary and use of drugs for which safer alternatives would be available. Safer alternatives could be chosen for alprazolam in anxiety, hydrochlorothiazide in preeclampsia and povidone iodine in respiratory tract infections.

In the drug utilization it is seen that average drug use per patient is 3.49. As it is obvious that hospitalized patients would receive higher number of drugs as compared in outpatients, we attempted to calculate drugs prescribed per patient among these two categories of patients. Our study showed that in an average 2.78 drugs per prescription were prescribed to out-patients. A similar study [12] shows an average of 1.66 ± 0.14 drugs per prescription among outpatients which is less as compared to our study. Taking into consideration that majority of prescriptions contained iron, calcium and folic acid, the number of drugs per patient is not a negative reflection which means in general fewer drugs were prescribed during pregnancy. This pattern is similar to other studies conducted which show use of iron, calcium and folic acid as the most frequently prescribed drugs during pregnancy [5, 12, 13, 25, 26] . Besides these, drugs for alimentary tract disorders, antiinfectives and nervous system disorders have also been frequently prescribed. A study [11] also shows analgesics (20 %), antibiotics (12.6 %), gastrointestinal system drugs (12.1 %), gyenocologic drugs (excluding antibiotics) (11.2 %) and central nervous system drugs (7.9 %) as the most frequently prescribed drugs.

As much as 85 % of the prescriptions were prescribed by brand names which shows that the practice of prescribing drugs at OBG ward of study site is predominantly brand name prescribing. Nearly 24 % of the drugs were fixed dose combinations which is significantly less as compared to previous studies where use of fixed dose combination was higher (64.8 %) [5].

The occurrence of complications and the need for drug use has always been a subject of interest when it is related with pregnancy. Although the study site was a single tertiary hospital, it is the major hospital of that region and the complications seen could be representative of the nature of complications occurring in Western Region of Nepal. Knowingly or unknowingly pregnant women are taking drugs and physicians prescribing them and in many instances when it goes wrong, the fetus is the sufferer. Because pregnant patients might not know about the safety profiles of the medicines prescribed to them, it is the responsibility of the prescriber to prescribe safe drugs during pregnancy. So, proper knowledge regarding medication use in pregnancy is highly essential before any drug is prescribed.

## Conclusion

A large number of complications during pregnancy were observed. Besides pain and nausea/vomiting, acid reflux disorders, upper respiratory tract infections, PV Bleeding, urinary tract infections were frequently encountered. The prescription of drugs to pregnant patients revealed the use of drugs almost only when necessary and those considered safe during pregnancy. However, few teratogenic drugs (2.49 % of total drugs prescribed) were also found to be prescribed which might need further assessments. More than 90 % of the pregnant patients received nutritional supplements of iron, calcium and folic acid.

## Limitations of the study

The study was completed in only one centre and so this data obtained from a tertiary care hospital cannot be accounted as a representative data from several primary and secondary care hospitals in Nepal. Also the study only analysed the drugs prescribed but not the outcomes of the therapy. So, the success of the therapy cannot be measured from this study.

## Additional files

Additional file 1: Association between complications and trimester of the respondents. (PDF 36 kb) Additional file 2: Drugs prescribed in observed complications. (PDF 49 kb) Additional file 3: Overall drugs per trimester. (PDF 47 kb)
