# Supplementary material for: Medication utilization pattern for management of pregnancy complications: a study in Western Nepal
Source: BMC Pregnancy Childbirth. 2016 Sep 20;16:272. doi: 10.1186/s12884-016-1068-8 (PMC5029070; doi:10.1186/s12884-016-1068-8)
Supplement: Additional file 1: — Association between complications and trimester of the respondents. (PDF 36 kb) [file 12884_2016_1068_MOESM1_ESM.pdf]

Additional File 1: Association between complications and trimester of the respondents

Complications	Trimester of the pregnancy			Total	p value
	First trimester	Second trimester	Third trimester		
Acid Reflux Disease	2 (10)	8 (40)	10 (50)	20 (100)	0.000
Anemia	1 (100)	0	0	1 (100)	
Anxiety	0	1 (50)	1 (50)	2 (100)	
Asthma	0	2 (66.7)	1 (33.3)	3 (100)	
Constipation	0	2 (50)	2 (50)	4 (100)	
Depression	0	1 (100)	0	1 (100)	
Diabetes Mellitus	0	1 (100)	0	1 (100)	
Diarrhea	1 (33.3)	1 (33.3)	1 (33.3)	3 (100)	
Dry eye	0	1 (100)	0	1 (100)	
Edema	1 (11.1)	3 (33.3)	5 (55.6)	9 (100)	
Epilepsy	1 (50)	0	1 (50)	2 (100)	
Fever/Headache	2 (20)	2 (20)	6 (60)	10 (100)	
Hyperemesis Gravidarum	5 (100)	0	0	5 (100)	
Hypothyroidism	1 (20)	0	4 (80)	5 (100)	
Irritable Bowel Disease	0	0	1 (100)	1 (100)	
Itching	0	2 (28.6)	5 (71.4)	7 (100)	
Loss of Appetite	0	2 (100)	0	2 (100)	
Loss of Pregnancy	7 (77.8)	0	2 (22.2)	9 (100)	
Nausea/Vomiting	15 (37.5)	17 (42.5)	8(20)	40 (100)	
Oligohydramnios	0	0	1 (100)	1 (100)	
Pain	0	15 (22.4)	52 (77.6)	67 (100)	
Pituitary Adenoma	0	0	1 (100)	1 (100)	
Pneumonia	0	0	1 (100)	1 (100)	
Polyhrdramnios	0	0	1 (100)	1 (100)	
Preeclampsia	0	0	6 (100)	6 (100)	
Psychosis	0	1 (100)	0	1 (100)	
PV Bleeding	5 (35.7)	0	9 (64.3)	14 (100)	
PV Discharge	0	0	8 (100)	8 (100)	
Thrombocytopenia	0	0	1 (100)	1 (100)	
Tingling Sensation	1 (50)	1 (50)	0	2 (100)	
Total	53 (19.3)	74 (26.9)	148 (53.8)	275 (100)	
Tuberculosis	1 (100)	0	0	1 (100)	
Upper respiratory tract infection	4 (16.7)	6 (25)	14 (58.3)	24 (100)	
Urinary Tract Infection	5 (26.3)	6 (31.6)	8 (42.1)	19 (100)	
Weakness	0	2 (100)	0	2 (100)	
