# Supplementary material for: Medication utilization pattern for management of pregnancy complications: a study in Western Nepal
Source: BMC Pregnancy Childbirth. 2016 Sep 20;16:272. doi: 10.1186/s12884-016-1068-8 (PMC5029070; doi:10.1186/s12884-016-1068-8)
Supplement: Additional file 2: — Drugs prescribed in observed complications. (PDF 49 kb) [file 12884_2016_1068_MOESM2_ESM.pdf]

Additional File 2 : Drugs prescribed in observed complications

Complication	Prescribed drugs
Abdominal Pain	Framycetin sulphate, hyoscine butylbromide, dexamethasone, isoxuprine, cefixime, ampicillin, ranitidine, ibuprofen + paracetamol
Acid Reflux disease	Ranitidine, antacids (aluminium + magnesium salts), pantoprazole, ondansetron, hyoscine butylbromide, rabeprazole, sucralfate
Anemia	Whole blood, iron, folic acid
Anxiety	Alprazolam
Asthma	Salbutamol, Ipratropium bromide, cefadroxil, oxygen
Back pain	Diclofenac, hyoscine butylbromide, paracetamol, amoxicillin, ranitidine
Constipation	Bisacodyl, lactulose, glycerin + sodium chloride, lignocaine, hyoscine butylbromide
Depression	Fluoxetine
Diabetes mellitus	Insulin
Dry eye	Polivinyl alcohol + sodium perborate
Edema	Diclofenac , ibuprofen + paracetamol, amoxicillin, hyoscine butylbromide, nifedipine , progesterone
Epilepsy	Carbamazepine, lamotrignine , valporic acid, phenobarbitone
Fever/ headache	Paracetamol, ibuprofen + paracetamol, azithromycin, amoxicillin, ceftriaxone, metronidazole, gentamicin, cefixime , ondansetron, ranitidine
Hyperemesis gravidarum	Ondansetron, metoclopramide, ranitidine, amoxicillin
Hypothyroidism	Thyroxine
Irritable bowel disease	Ranitidine, antacids (aluminium + magnesium salts)
Itching	Loratidine, amoxicillin, Clotrimazole, L arginine +Zinc sulphate + folic acid combination, Calamine + zinc oxide
Loss of pregnancy	Misoprostol, tranaxemic acid, cefixime, metronidazole, azithromycin, secnidazole, hysocine butylbromide, ibuprofen + paracetamol, mefinamic acid, progesterone, hydroxyprogesterone, HCG
Lower abdominal pain	Hyoscine butylbromide, diclofenac, ibuprofen + paracetamol, alendronate
Nausea/ vomiting	Ondansetron, domperidone, granisetron, metoclopramide, ranitidine, pseudroephedrine + chlorphenarimine + paracetamol, cefixime
Neck pain	Diclofenac
Oligohydraminos	Ceftriaxone, metronidazole, ranitidine, ibuprofen + paracetamol
Perianal itching	Mebendazole, cetirizine
Pneumonia	Terbutaline + bromohexidine + loratidine, hyoscine butylbromide, paracetamol, piperacillin + tazobactam, amoxicillin + potassium clavulanate
Polyhydraminos	Amoxicillin, ibuprofen + paracetamol, ranitidine
Preeclampsia	Methyldopa, nifedipine, amlodipine, hydrochlorothiazide, magnesium sulphate, pyridoxine, bromocriptine, aspirin
Psychosis	Olanzepine
PV Bleeding	Iron, folic acid, progesterone, tranaxemic acid, dexamethasone, cefixime, ceftriaxone, metronidazole, methylergotamine, blood transfusion, ibuprofen + paracetamol, diclofenac, hyoscine butylbromide
PV discharge	Metronidazole + clotrimazole +lactobacillus, ceftriaxone, metronidazole, Hyoscine butylbromide, clotrimazole, diclofenac, betamethasone
Thrombocytopenia	Iron, calcium and folic acid
Tuberculosis	Isoniazid, rifampin, pyrazinamide, ethambutol
Urinary tract infection	Paracetamol, hyoscine butylbromide, fexofenadine, ibuprofen + paracetamol, bromhexine+pseudoephrine+chlorphenarimine, azithromycin, amoxicillin, ceftriaxone, cefixime, cephazolin, povidone iodine, loratidine, nitrofurantoin,ampicillim, gentamicin, piperacillin + tazobactam, framycetin sulphate, flavoxate
Upper respiratory tract infections	Povidone iodine gargle, loratidine, paracetamol, terbutaline + bromohexidine, cetirizine, levocetirizine, paracetamol, oxymetazoline, terbutaline + bromhexidine + guanefesin, chlorhexidine, domperidome, chlorphenarimine, amoxicillin, azithromycin, cefixime, Dextromethorphan + Chlorphenaramine + Phenylephrine

Note: Here major drugs prescribed for a particular complication are shown. Complications for which drugs have not been prescribed are not included. Iron, calcium, folic acid, vitamins, tetanus toxoid vaccine given as routine supplementation are not included.
