# Supplementary material for: Medication utilization pattern for management of pregnancy complications: a study in Western Nepal
Source: BMC Pregnancy Childbirth. 2016 Sep 20;16:272. doi: 10.1186/s12884-016-1068-8 (PMC5029070; doi:10.1186/s12884-016-1068-8)
Supplement: Additional file 3: — Overall drugs per trimester. (PDF 47 kb) [file 12884_2016_1068_MOESM3_ESM.pdf]

Additional file 3: Overall drugs per trimester

Category	Drug	1st Trimester	2nd Trimester	3rd Trimester	TOTAL
**Alimentary Tract and Metabolism**	Aluminium Hydroxide + Magnesium Hydroxide	0	1	3	4
	Bisacodyl	0	1	0	1
	Calcium	9	57	133	199
	Domperidone	0	2	0	2
	Glycerine + Sodium Chloride	0	0	1	1
	Granisetron	0	0	1	1
	Hyoscine Butylbromide	2	6	26	34
	Insulin Human	0	1	0	1
	Lactulose	0	0	1	1
	Magnesium Sulphate	0	0	1	1
	Metoclopramide	4	0	0	4
	Ondansetron	12	10	6	28
	Pantoprazole	0	0	3	3
	Pyridoxine	0	2	0	2
	Rabeprazole	0	0	1	1
	Ranitidine	11	12	30	53
	Sucralfate	0	0	2	2
	Vitamin B Complex	6	2	2	10
	Vitamin C	0	0	1	1
**Anti-infectives for Systemic Use**	Amoxicillin	6	2	20	28
	Amoxicillin + Potassium Clavulanate	0	1	3	4
	Ampicillin	1	0	0	1
	Azithromycin	2	3	2	7
	Cefadroxil	0	1	0	1
	Cefixime	10	3	18	31
	Ceftriaxone	4	3	11	18
	Cephazolin	0	1	0	1
	Ethambutol	1	0	0	1
	Gentamicin	0	0	2	2
	Isoniazid	1	0	0	1
	Metronidazole	5	1	15	21
	Nitrofurantoin	0	1	0	1
	Piperacillin + Tazobactam	1	0	1	2
	Pyrizinamide	1	0	0	1
	Rifampin	1	0	0	1
	Tetanus Toxoid Vaccine	1	10	11	22
**Antiparasitic Products, insecticides and Repellants**	Mebendazole	0	0	1	1
	Secnidazole	1	0	0	1
**Blood and Blood Forming Organs**	Blood for Transfusion (Whole)	2	0	0	2
	Folic Acid	24	10	8	42
	Iron	3	8	25	36
	Iron + Folic Acid Combination	11	51	111	173
	Tranexamic Acid	2	0	1	3
**Cardiovascular System**	Amlodipine	0	0	3	3
	Hydrochlorothiazide	0	0	1	1
	Isoxsuprine	0	0	2	2
	Methyldopa	0	0	6	6
	Nifedipine	0	0	7	7
**Dermatologicals**	Calamine + Zinc Oxide	0	0	1	1
	Framycetin Sulphate	0	0	2	2
	Lignocaine	0	1	0	1
	Povidone Iodine	0	2	0	2

Genitourinary System and Sex Hormones	Clotrimazole	0	2	2	4
	Flavoxate	0	0	1	1
	HCG	1	0	0	1
	Hydroxyprogesterone	1	0	0	1
	Methylergometrine	0	0	1	1
	Misoprostol	3	0	2	5
	Progesterone	8	0	1	9
Muscoloskeletal System	Alendronate	0	0	1	1
	Diclofenac	0	2	8	10
	Mefinamic acid	1	0	0	1
Nervous System	Alprazolam	0	0	2	2
	Aspirin	0	0	1	1
	Bromocriptine	0	0	3	3
	Carbamazepine	1	0	0	1
	Fluoxetine	0	1	0	1
	Ibuprofen + Paracetamol	4	0	26	30
	Lamotrignine	0	0	1	1
	Olanzapine	0	1	0	1
	Paracetamol	8	8	18	34
	Phenobarbitone	0	0	1	1
	Pseudroephrine + Chlorphenarimine +Paracetamol	0	1	0	1
	Sodium valporate + Valporic acid	0	0	1	1
Respiratory System	Bromohexine + Pseudoephrine + Chlorphenarimine	0	1	0	1
	Cetirizine	1	2	5	8
	Chlorhexidine	0	0	1	1
	Chlorpheniramine	0	0	1	1
	Dextromethorphan + Chlorphenaramine + Phenylephrine	2	1	4	7
	Fexofendine	0	3	0	3
	Ipratropium Bromide	0	1	0	1
	Levocetrizine	0	1	0	1
	Loratadine	2	1	3	6
	Oxymetazoline	1	1	1	3
	Salbutamol	0	2	0	2
	Terbutaline + Bromohexedine + Guanefesin	0	1	1	2
Sensory Organs	Polivinyl alcohol + Sodium perborate	0	1	0	1
Systemic Hormonal Preparations exc Sex Hormones and Insulin	Betamethasone	0	0	1	1
	Dexamethasone	0	0	7	7
	Thyroxine	1		4	5
Various	Alternative Medicines	0	3	4	7
	L- Arginine + Zinc Sulphate + Folic Acid	0	0	1	1
	Metronidazole + Clotrimazole + Lactobacillus	0	0	2	2
	Nutritional Supplements	5	1	8	14
	Oxygen	0	0	1	1
